# The mitohormetic response as part of the cytoprotection mechanism of berberine: Berberine induces mitohormesis and mechanisms

**DOI:** 10.1186/s10020-020-0136-8

**Published:** 2020-01-23

**Authors:** Xiaofei Zhu, Yihui Wei, Beibei Yang, Xiaoxiao Yin, Xiaofang Guo

**Affiliations:** 1 Department of Clinical Immunology, School of Laboratory Medicine, Xinxiang Medical University, Xinxiang, 453003 China; 2 Henan Collaborative Innovation Center of Molecular Diagnosis and Laboratory Medicine, Xinxiang Medical University, Xinxiang, 453003 China; 3 Henan Key Laboratory of Immunology and Targeted Drugs, Xinxiang Medical University, Xinxiang, 453003 China; 4 Department of Microbiology, School of Basic Medical Sciences, Xinxiang Medical University, Xinxiang, 453003 China

**Keywords:** Berberine, Mitohormesis, Reactive oxygen species, Nicotinamide adenine dinucleotide, Mitochondrial unfolded protein response

## Abstract

It was well-known that Berberine, a major bioactive compound extracted from natural plants *Coptis chinensis*, has anti-diabetic effects for decades in china. Other types of pharmacological activities, such as anti-inflammatory, antimicrobial, hypolipidemic, and anti-cancer effects, have also been examined. At cellular level, these pharmacological activities were mostly an inhibitory effect. However, the cytoprotective effect of berberine was also observed in various types of cells, such as neurons, endothelial cells, fibroblasts, and β-cells. The paradoxical result may be closely associated with characteristics and distribution of berberine within cells, and they can be explained mechanically by mitohormesis, one particular form of hormesis. Here, we reviewed the mitohormetic response and assessed the berberine-induced effects and the possible signaling pathway involved. These findings may contribute to better clinical applications of berberine and indicate that some mitochondria-targeted conventional drugs should be considered carefully in clinical application.

## Introduction

At a cellular level, mitochondria play a critical role in cell’s adaptation to external stressors, such as chemical toxicants, xenobiotics, and pathogens. These potentially damaging stressors could induce mitochondrial stress response by targeting pathways directly or indirectly involved in energy production and signaling required for survival. When the strength of stressors exceeded the adaptive capacity of cells, it could cause mitochondria-mediated cell death (Valera-Alberni and Canto 2018; Lan et al. 2019). In toxicology, this biphasic dose response, called as “hormesis,” was observed in many natural active ingredients from traditional Chinese medicine (Wang et al. 2018; Liu et al. 2019). This phenomenon indicated that conventional “toxic” drugs may have beneficial effects on cells. Evidence from both in vivo and in vitro studies has indicated that mild or sublethal mitochondrial stress from chemicals, especially some mitochondria-targeted drugs, showed beneficial effects on cells and organisms against larger subsequent stresses-induced damages or death (Cox et al. 2018; Obata et al. 2018; Yuyun et al. 2012; De Haes et al. 2014). This response activated by a moderate mitochondrial stress has been named mitohormesis, and it can maintain cellular homeostasis and extend lifespan (Tapia 2006; Yun and Finkel 2014).

Berberine (Ber) is a botanical alkaloid isolated mainly from the root and bark of several plants, such as *Coptidis rhizoma* and *Hydrastis canadensis*. According to the ancient records of traditional Chinese medicine listed in *The Divine Farmer’s Classic of Materia Medica* (*Shen Nong Ben Cao Jing*), *Coptidis rhizoma* can be used to treat dysentery and diarrhea. Ber is one of the main active ingredients in *Coptidis rhizoma*, accounting for 5.2–7.7% (Huang and JNM 1986; Berberine 1991). Clinical trials revealed that Ber also exhibits antimicrobial and anti-inflammatory activities in infectious diseases. It is a non-prescription drug used to treat gastrointestinal infections in China (Qu 2006). Moreover, other pharmacological effects of Ber, such as anti-diabetic, anti-obesity and anti-cancer, have been also unravelled (Yan et al. 2017; Pang et al. 2015; Kong et al. 2004). However, there were conflicting results reported in the literature that opposite effects of Ber exhibited in different type of cells, such as protective effect on neuronal cells (Zhang et al. 2017) or apoptosis-induced effect on cancer cells (Bao et al. 2015; Yan et al. 2017). Even in same type of cells, for example, cancer cells, it was also found that the effects of Ber was opposite. These phenomena were discussed and attributed to hormetic effect of Ber (Bao et al. 2015). But in these studies, a common results was demonstrate that Ber at low dose could exerted a cytoprotection effect in all types of cells, including cancer cells (Bao et al. 2015; Gao et al. 2011; Guo et al. 2016; Yan et al. 2017; Zhu et al. 2017).

Actually, mitohormesis is a biological response activated by a potentially external stressors in mitochondria. The mitochondrial stress response leads to an improvement in diseases and health and viability within a cell via mitonuclear communication (Yun and Finkel 2014). The mechanism of this interplay between mitochondria and nuclear were involved in a broad and diverse cytosolic and nuclear signalling pathways, including reactive oxygen species (ROS) (Ristow 2014), the mitochondrial unfolded protein response (UPRmt) (Jovaisaite et al. 2014), and mitochondrial metabolites (Toyama et al. 2016; Canto et al. 2015). As an important target and a major subcellular localization of Ber at low dose (Serafim et al. 2008; Pereira et al. 2007), mitochondria play a key role in activity of Ber (Yan et al. 2017). This may be partly explained the different effect in different energy-demanded cells, such as cancer cells, which may be associated with different sensitivities to Ber. As similar to functions of another mitochondrial-targeted drug metformin (Wang et al. 2017), Ber could induce mitochondrial stress response against stress-induced cellular damage through multiple pathways, such as mitochondrial respiratory chain-mediated ROS production (Turner et al. 2008; Lenaz 2001) and Nrf2 signalling pathway (Zhang et al. 2017; Jiang et al. 2019), AMPK signalling pathway (Turner et al. 2008). These pathways also crosstalk with mitonuclear communication signalling pathways in mitochondrial stress response. Here, the possible cytoprotection mechanism of Ber via mitohormesis were reviewed.

### Cellular uptake and subcellular location of Ber

Ber is a hydrophilic compound with high solubility in basic solution and low permeability. Under physiological conditions, it mainly exists in a positively charged protonated form. In normal water solution, only a few Ber particles are converted to aldehyde or alcohol-type, and these possess lipophilic properties. In this way, it was difficult for Ber to rapidly and passively diffuse through cell membranes (Berberine 1991; Zhang et al. 2014). However, as a substrate of organic cation transporter 1 (OCT1, *SLC22A1* gene) and organic cation transporter 2 (OCT2, *SLC22A2* gene), Ber could be taken up into cells at a relatively fast rate (Nies et al. 2008; Shi et al. 2018). OCT2 is also expressed in the central nervous system. This may explain how Ber can penetrate the blood-brain barrier and so play a protective role in neurons (Sun et al. 2014). In living cells, the Ber first accumulated on the mitochondria due to its physicochemical properties. As the amount of uptake increased, Ber could accumulate in the cytoplasm or nucleus, possibly because of saturation in the mitochondria. The subcellular location may partially explain the paradoxical results in cell fate (Serafim et al. 2008; Mikes and Dadák 1983). For example, it has recently been reported that Ber at low dose range (1.25–5 μM) could promote cancer cell proliferation and significantly attenuate the anticancer activity of chemotherapeutic agents in combination drug regimens (Bao et al. 2015).

### Pathway of berberine-induced mitohormetic response

#### ROS signaling pathways

The mitochondria are not only the main powerhouse of bioenergy but also a source of ROS. The majority of ROS are products of the mitochondrial respiratory chain, especially at the site of respiratory chain complex I and III (Turrens 2003). However, an increase in ROS did not mean that it was harmful to cell survival. Several studies have shown that, under physiological conditions, as signalling molecules, the transient increase in ROS could induce some transcriptional changes in the nucleus by mitohormetic response to regulate cell adaption to an unfriendly environment (Obata et al. 2018; Zarse et al. 2012; Ristow 2014).

Ber could inhibit mitochondrial respiration by targeting complex I (Turner et al. 2008), which led to leakage of electrons that cause a higher rate of reactive oxygen production in the mitochondria (Lenaz 2001). ROS could transduce signals to the nucleus by triggering the oxidation of several reactive Cys residues in redox-dependent manner (Truong and Carroll 2012). The redox modification of proteins could translocate to and accumulate in the nucleus to induce host-antioxidant defense genes, such as the mammalian Kelch-like ECH-associated protein 1 (KEAP1)–nuclear factor erythroid 2-related factor 2 (NRF2) (Taguchi et al. 2011). Ber was also proposed as a potential anti-aging agent (Zhao et al. 2013) and exhibited a neuroprotective effect via the ROS-meditated pathway (Zhang et al. 2017). In this way, a transient rise in ROS levels induced by a low dosage of Ber may protect cells through a potential feedback mechanism involved in anti-oxidative defence or stress defence pathways, such as Nrf2 signaling pathway, to resist larger subsequent stress-induced damage (Jiang et al. 2019).

#### Metabolite signaling pathways

Adenosine triphosphate (ATP) is an important metabolite produced by mitochondria through oxidative phosphorylation (OXPHOS). Decreases in ATP levels can increase the ratio AMP/ATP and activate the adenosine monophosphate (AMP) sensor, the AMP-activated protein kinase (AMPK), which is a master regulator of cellular metabolism. The phosphorylated-activation of a downstream signaling pathway via AMPK can enhance mitochondrial energy harvesting by decreasing ATP consumption (Herzig and Shaw 2017), and maintain mitochondrial homeostasis by promoting mitophagy and mitochondrial fission (Egan et al. 2011; Toyama et al. 2016). Ber could active AMPK pathway by inhibiting mitochondrial respiration, which increased the ratio of AMP/ATP (Turner et al. 2008). Pharmacological activation of AMPK by Ber had protective effects against cellular senescence and apoptosis and exhibit therapeutic efficacy in metabolic and neurodegenerative conditions as well as and other aging-related diseases (Zhang et al. 2017; Han et al. 2016; Wang et al. 2011; Zhao et al. 2014).

Nicotinamide adenine dinucleotide (NAD^+^) is also an important metabolite. As a key cofactor of multiple dehydrogenases, the levels of NAD^+^ and the ratios of NAD^+^/NADH are primarily maintained by mitochondria via the tricarboxylic acid (TCA) cycle and OXPHOS function. During energy deficits, NAD^+^ levels become elevated, which can be protective against disease and increase lifespan in mice (Canto et al. 2015; Zhang et al. 2016). NAD^+^ is also an essential co-substrate of sirtuins, such as SIRT1, which promoted mitochondrial biogenesis, and its function was closely associated with lifespan (Imai and Guarente 2014). Therefore, elevation of NAD^+^ levels by medication may be an effective strategy for aging-related diseases (Houtkooper and Auwerx 2012). Ber may induce an increase of intracellular NAD^+^ levels by moderately inhibiting OXPHOS, which was similar to energy deficits (Turner et al. 2008; Yin et al. 2008). Ber may also increase intracellular NAD^+^ concentrations indirectly through AMPK activation, so regulating the expression and activity of nicotinamide phosphoribosyl transferase (NAMPT), a key rate-limiting enzyme in NAD^+^ synthesis, which could increase sequential SIRT1 activity (Brandauer et al. 2013; Cantó et al. 2009).

#### Unfolded protein response signaling pathways

The mitochondrial unfolded protein response (UPRmt) is a stress response pathway that maintains mitochondrial homeostasis, specifically proteostasis. The UPRmt could be induced by intramitochondrial damage. Its activation has been shown to elicit a retrograde signaling pathway from mitochondria to the nucleus, which results in the expression of proteases, chaperonins, and other stress response genes to restore mitochondrial protein homeostasis. Multiple stressors have been shown to be involved in the activation of UPRmt, such as perturbation of OXPHOS, impairment of mitochondrial ribosomes, and high levels of ROS (Jovaisaite et al. 2014; Zhao et al. 2002).

UPRmt can be pharmacologically activated by antibiotics, such as tetracyclines and phenicols, in living organisms (worms, flies, and mammals), which leads to UPRmt-dependent increases in longevity and health span (Quiros et al. 2016; Moullan et al. 2015). It has also been reported that this pharmacological treatment can have protective effects in several neuromuscular disorders, such as amyotrophic lateral sclerosis and Guillain-Barré syndrome (Zhu et al. 2002; Zhang et al. 2009). Ber could accumulate not only in mitochondria to influence energy metabolism but also bind to DNA or RNA to regulate gene expression (Yuan et al. 2015). This may be closely associated with the dosage used at the cellular level (Yan et al. 2017). At low doses, Ber may disturb OXPHOS and bind to mtDNA to activate UPRmt, which may contribute to cytoprotective effects, whereas it was reverse at high dose (Bao et al. 2015; Yan et al. 2017; Turner et al. 2008; Bhadra et al. 2008).

In addition, elevation of NAD^+^ levels may activate the UPRmt in both mammals and nematodes partly through NAD^+^-driven activation of SIRT1 (Zhang et al. 2016; Mouchiroud et al. 2013; Gariani et al. 2015). Elevated NAD^+^ levels and overexpression of SIRT1 robustly increased the protein levels of the mammalian UPRmt homolog Hsp60 and UPRmt protease CLpP (Khan et al. 2014; Calabrese 2008). This may be how Ber exerts its cytoprotective effects by activating the UPRmt through Ber-mediated elevation of NAD^+^ levels or upregulation of SIRT1 expression (Zhu et al. 2017; Turner et al. 2008; Yin et al. 2008).

## Conclusion

Hormesis is a biphasic dose response to a chemical agent, which was first proposed and used in the toxicology field. In biology, it also means to an adaptive response activated by a low dose of stress stimuli, such as caloric restriction and phytochemicals, in cells and organisms to maintain homeostasis, whereas it has a harmful effect at higher doses (Calabrese 2008; Mattson 2008). The mitochondria are key to nutrient metabolism and bioenergy production and essential to cellular homeostasis. It was proposed and supported experimentally that sublethal mitochondrial stress should cause a beneficial hormetic response called mitohormesis (Wang et al. 2018; Obata et al. 2018). As a bioactive component from traditional Chinese medicine, Ber showed a protective effect on cells in harsh environments, which was associated with a mitohormetic response (Serafim et al. 2008; Bao et al. 2015; Gao et al. 2011; Guo et al. 2016; Yan et al. 2017; Zhu et al. 2017).

**Fig. 1:**
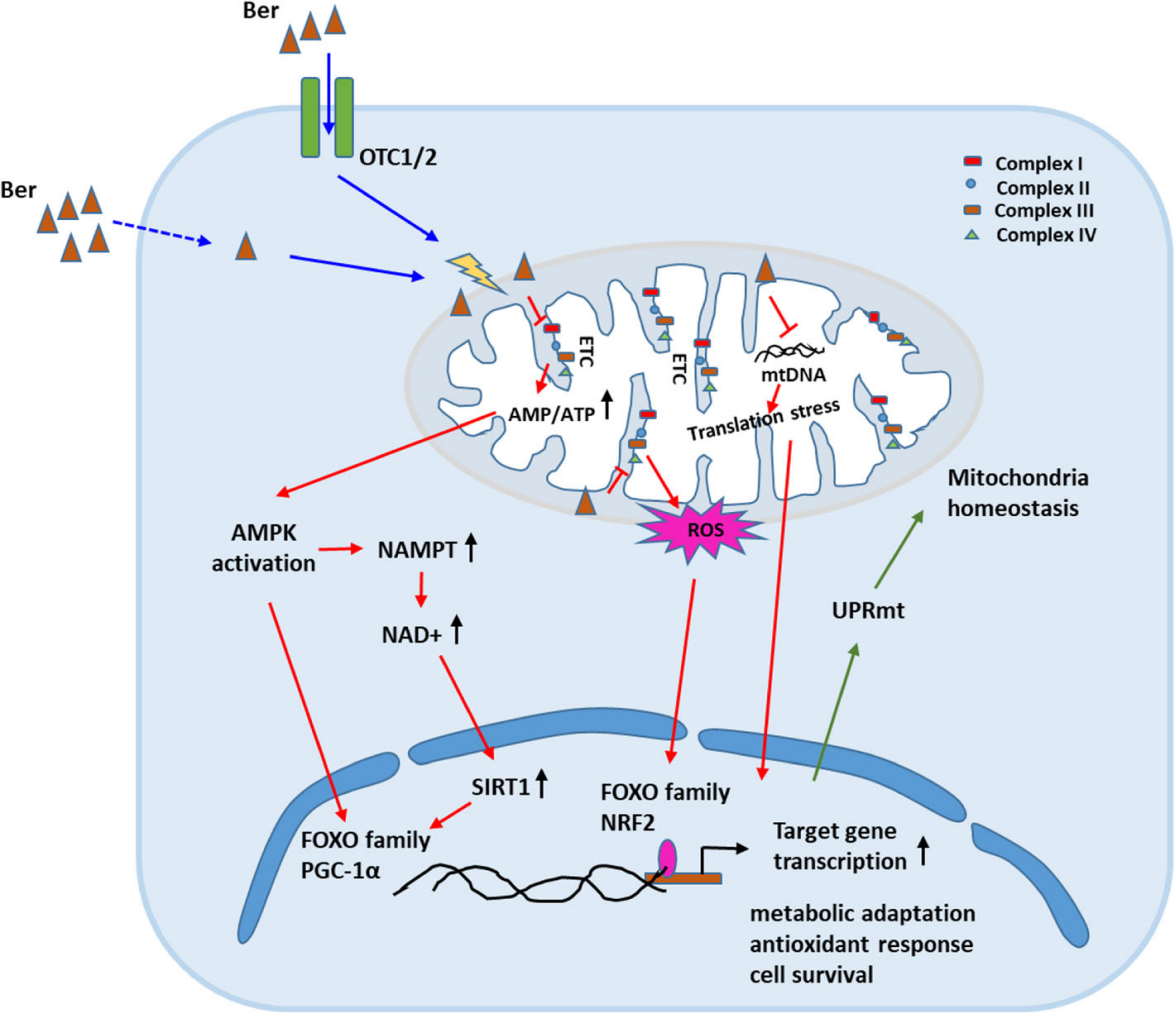
Overview of Ber-mediated mitohormesis signaling. Ber mainly accumulated in the mitochondria after entering cells through organic cation transporter (OTC1/2) or passive diffusion. In mitochondria, Ber could target enzymes and other proteins associated with the electron transfer chain or mtDNA to disrupt energy homeostasis and induce translation stress. This can induce mitohormetic response via (1) ROS-mediated redox pathway, (2) AMP/ATP-induced AMPK pathway, (3) NAD+/NADH-mediated Sirtuins pathway (i.e., SIRT1), and (4) UPRmt pathway to regulate and maintain mitochondria homeostasis for the ability of cells to adapt to adverse circumstances

Here, we review the current understanding of possible retrograde signaling pathways involved in berberine-meditated mitohormesis. A low dose of Ber could target mitochondria through the physicochemical properties of its positively charged form. Ber mildly inhibited electron transport chain (ETC) by accumulating in mitochondria and causing a decrease in the efficiency of energy produced of OXPHOS (i.e., ATP) and a moderate increase of ROS and NAD^+^ (Turner et al. 2008; Lenaz 2001; Yin et al. 2008; Brandauer et al. 2013; Cantó et al. 2009). This could lead to a mitohormetic response in the following signaling pathways: (1) ROS-mediated redox pathway, (2) AMP/ATP-induced AMPK pathway, (3) NAD^+^/NADH-mediated Sirtuins pathway (i.e., SIRT1), and (4) UPRmt pathway. In a sense, upstream of these pathways originated from energy stress (ATP deficits), and signal interactions existed downstream of these pathways, such as AMPK-regulated NAD^+^ increase (Brandauer et al. 2013; Cantó et al. 2009) and SIRT1-regulated UPRmt-relative gene expression (Mouchiroud et al. 2013) (Fig. 1). All of these pathways could ultimately enhance the adaptiveness of cells to adverse circumstances by upregulating transcription involved in resolving metabolic adaptation, the antioxidant response, and cell survival.

As important intracellular organelle of nutrient and energy metabolism, mitochondria have an essential role in controlling the fate of cells, such as cell death and immunity (Mehta et al. 2017; Orrenius et al. 2007). In this way, mitochondria are central platforms to support cell function and maintain cell homeostasis. Given probable mitohormetic effect of drug related to low dosage, some mitochondria-targeted conventional drugs should be interrogated dialectically in clinic applications, such as statins (Marcheggiani et al. 2019; Bouitbir et al. 2012). Moreover, mitochondria-targeted agents, such as rotenone and metformin, also exhibit protective effects on cellular survival and extending lifespan at a low concentration via ROS-mediated mitohormetic signaling pathways (Yuyun et al. 2012; De Haes et al. 2014). This implied that mitochondria-targeted agents may produce a beneficial effect in aging-relative diseases via mitohormesis (Marcheggiani et al. 2019; Bouitbir et al. 2012; Liu et al. 2019).

## Data Availability

Not applicable.

## Abbreviations

AMP: Adenosine monophosphate
AMPK: AMP-activated protein kinase
ATP: Adenosine triphosphate
Ber: Berberine
ETC: Electron transport chain
FOXO: Forkhead box protein O
NAD: Nicotinamide adenine dinucleotide
NAMPT: Nicotinamide phosphoribosyl transferase
OCT: Organic cation transporter
OXPHOS: Oxidative phosphorylation
ROS: Reactive oxygen species
SIRT1: Sirtuin 1
TCA: Tricarboxylic acid
UPRmt: mitochondrial unfolded protein response
